# Life and Death: Some Human Lessons in War-Time

**Published:** 1916-10-14

**Authors:** 


### THE EDITOR'S LETTER-BOX.

### Life and Death: Some Human Lessons in War-Time.

### SUGGESTION AND FAITH.

*To the Editor of* THE HOSPITAL.

SIR,—It is with keen interest that I welcome the series of articles now appearing in THE HOSPITAL under the head of "Life and Death," for they raise the whole question of mental and spiritual healing. Of course, at the present time the medical profession has accepted psychotherapy as a line of treatment that should have a recognised place in our therapeutic armoury. But it is also a fact—to my mind an unfortunate fact—that medical men, after refusing for many years to listen to the claims of hypnotic suggestion, have lately been inclined to welcome the phenomena of suggestion as explaining finally the variety of psychological happenings that have otherwise been interpreted as being evidence of man's relationship with a spiritual sphere.

Now, whilst hypnotic suggestion in its ordinary application is undoubtedly an invaluable aid to the treatment of many functional conditions, it would surely be absurd to accept the method as the be-all and end-all of psychotherapy; indeed, any medical psychologist of experience knows that the system of direct suggestion does not go very far to help us with the great mass of persons obsessed by what the late Professor William James well termed "sick souls"; and in my opinion the same applies to the more modern method of psycho-analysis. In psychotherapeutic training I think that the understanding and study of the technique associated with the therapeutic application of hypnotic suggestion and psycho-analysis are an important step in the practical training of the medico-psychologist, but it would be a great mistake to confine our psychotherapeutic efforts to these methods. The point I wish to emphasise is this: that whilst admitting that treatment by suggestion, and possibly psycho-analysis, should have an important place in scientific therapy, we must look beyond if we are to succour and to give really valuable assistance to the huge army of mentally troubled persons who are now resorting to the shrines of various spiritual healers. Instead of scoffing at the practical methods of such healers, we might at least carefully investigate the general principles running through the technical procedures of various schools of metaphysical—so-called "mental and spiritual"—healing which are undoubtedly believed in by very honest and worthy people, and which undoubtedly seem to give successful results in the relief of many ailments. And it is the relief afforded by such principles which as a matter of practical experience seem to take us farther than the efforts of the orthodox physician practising hypnotic suggestion, that must make us give really serious consideration to this point. Clearly I do not advocate that we should deny the relative existence of matter or disease, and tell our appendicitis patients that their trouble is merely due to mental error, neither do I advise the treatment of the wounded by the simple affirmation of their wholeness and the discarding of splints and dressings, but I do advocate that medical practitioners should realise that when they have done all that is possible on the physical side they should remember that patients have feelings and spiritual characteristics which can be so influenced as to increase their natural energies and powers of recovery.

As a practical consideration it would appear that a great many people have allowed themselves to get into materialistic habits of thought, forgetting that healthy man, with few exceptions, requires an optimistic spiritual philosophy to round off his ordinary life. Without going into regions of religion and abstruse metaphysics, I would point out that medical men do not sufficiently realise what a powerful lever for successful psychotherapeutics is to be found in the mere declaration and reminder that a sufferer is not to forget his innate spiritual nature nor his spiritual inheritance. And, as a matter of fact, it is just some declaration of this kind that is the first step taken by many "spiritual healers."

Of course, when one regards the problem of scientific psychotherapy from this point of view, it is at once apparent that, when one has to deal with the mind of man, simple suggestion, as a healing medium, either with or without hypnosis, can only take us part of the way—it may be said, indeed, it can take us as far as it is our duty as medical practitioners to go. But to say this is to admit the contention that there is an advanced stage of psychotherapeutic procedure which must be left in other hands; which is just what we ought to avoid.

In spite of the recent tendencies to materialistic thought, most people have, after all, within themselves a longing to get into touch with some higher form of being, and do not find it difficult to assimilate ideas of a spiritual future, of a higher spiritual present, and of spiritual powers capable of helping them in everyday routine, especially when such thoughts are presented to them by a strong, clear thinker who himself has no doubt of their truth. Such a psychotherapeutic cure and upliftment may be brought about by a doctor whose life is based on spiritual understanding, by a clergyman whose advice has been sought, and who happens to be in touch with the modern mind-cure movement, or by what we term an irregular practitioner, who, as a means of professional livelihood, treats people on metaphysical lines. But whoever brings about the cure in this way certainly at the same time lifts up the invalid's whole life. Therefore I earnestly appeal to the medical profession for their immediate consideration of a wider psychotherapy than has so far been admitted as a reasonable adjunct to ordinary routine treatment.—I am, faithfully yours,

Harley Street, W., Oct. 6, 1916. E. L. A.

